# Immuno-modulatory Effect of IFN-gamma in AMD and its Role as a Possible Target for Therapy

**DOI:** 10.4172/2155-9570-S2-007

**Published:** 2013-02-26

**Authors:** Kailun Jiang, Sijia Cao, Jing Z Cui, Joanne A Matsubara

**Affiliations:** 1 Department of Ophthalmology and Pathology and Laboratory Medicine, University of Ottawa, Ottawa, Canada; 2 Department of Ophthalmology and Visual Sciences, Faculty of Medicine, University of British Columbia, Vancouver BC, Canada

**Keywords:** Age-related macular degeneration, Interferon gamma, Cytokines, Pro-inflammatory modulation, RPE, Drusen, STAT1, CXCL11

## Abstract

Age-related macular degeneration (AMD) is a neurodegenerative disease characterized by retinal cell atrophy, and/or choroidal neovascularization in the macula and constitutes the most common cause of blindness among the elderly in industrialized countries. The management of AMD is constrained by our insufficient knowledge of its underlying mechanisms. Recent studies point towards an emerging involvement of interferon-gamma (IFN-γ), a soluble cytokine associated with innate and adaptive immunity. IFN-γ promotes proinflammatory responses by activating proinflammatory cytokines and chemokines, thereby recruiting immune cells such as macrophages and T cells. On the other hand, IFN-γ modulates inflammatory response by upregulating anti-inflammatory factors or inhibiting development of immune cells related to autoimmune response. The complex role of IFN-γ in AMD pathogenesis is intriguing and worth further investigation in terms of therapeutic development.

## Introduction

Age-related macular degeneration (AMD) is the most common cause of blindness among the elderly in industrialized countries [1]. AMD is characterized by retinal pigment epithelium (RPE) dysfunction and sub-RPE drusen formation in the early stage. With time, it may progress to retinal cell atrophy, and/or choroidal neovascularization in the macula. Recent studies incorporating genetic and epidemiological data have made a credible argument for chronic inflammatory events playing a central role in the pathogenesis and development of AMD. Interferon-gamma (IFN-γ), a soluble cytokine associated with innate and adaptive immunity, is considered to be a pro-inflammatory factor. Recent studies point towards an emerging relationship between IFN-γ and mechanisms underlying the pathogenesis of AMD. Along with other pro-inflammatory factors such as IL-1 and TNF-α, IFN-γ functions synergistically to activate inflammatory components, including the complement cascade and recruit immune cells such as macrophages, microglia, NK and T cells [2–5]. In AMD eyes, these immune cells are present in areas surrounding the outer retina and drusen deposits [6–8], they can induce direct damage to photoreceptors [8,9], potentially leading to vision loss. Yet, the interaction of pathways activated by IFN-γ is complex and not fully understood. Also, the role of IFN-γ as a possible therapy target is still unclear. Herein, we will review the literature on IFN-γ in the outer retina with focus on its role as a potential target for therapy for chronic inflammatory diseases of the eye.

## Is IFN-γ a Possible Target for Treatment of AMD?

**Figure 1:**
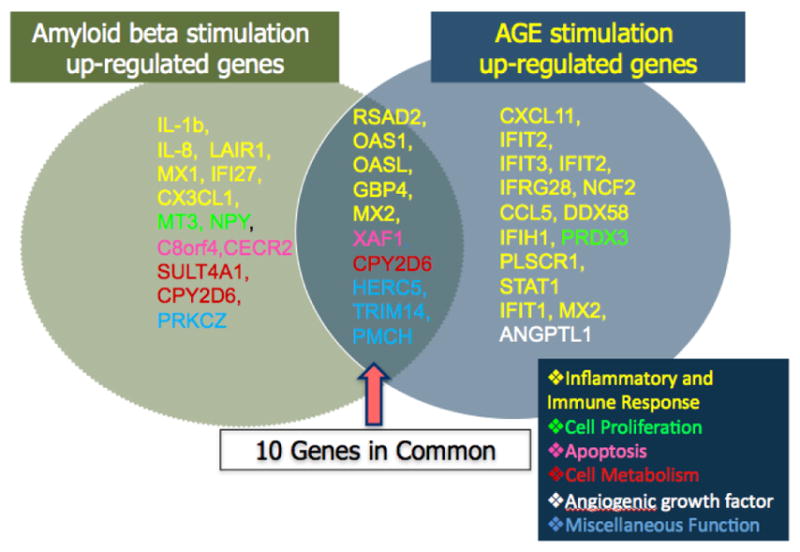
Summary diagram of differentially expressed gene results obtained from a microarray study of human RPE cell response to *in vitro* stimulation with amyloid beta (0.3 μM, left oval) or advanced glycation endproducts (AGE, 10 μg/mL, right oval). Amyloid beta and AGE are two known components of drusen, and results suggest that both induce proinflammatory responses, including IFN-γ signaling.

**Figure 2:**
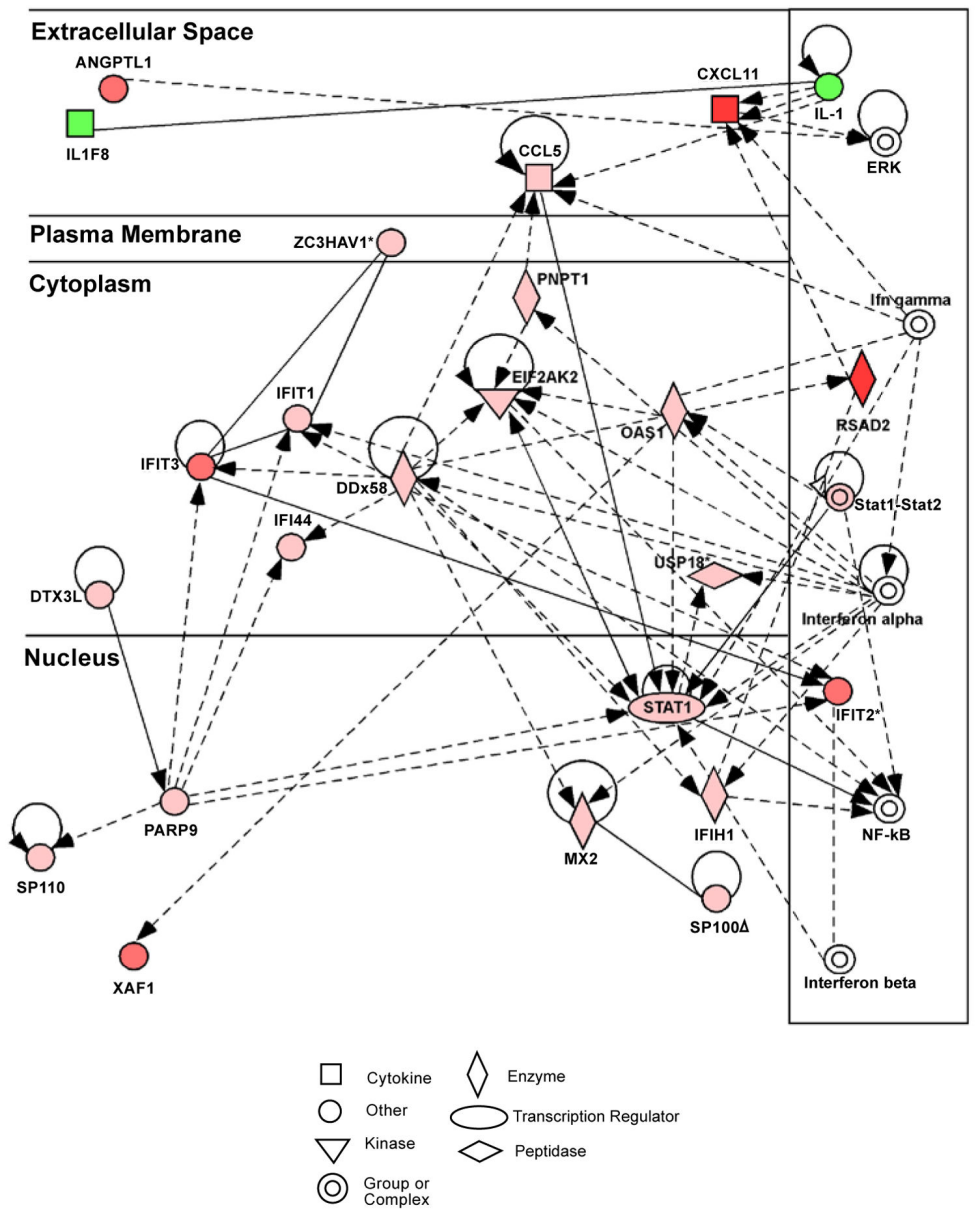
Molecular network generated by Ingenuity Pathway Analysis (IPA) of highly significant gene changes in human RPE cells after *in vitro* stimulation with AGE (10 μg/mL). Colored symbols represent genes that were significantly highly upregulated (red) with decreasing relative levels indicated by lighter shades (pink and light pink) or downregulated (green) in our data set [11]. The white entries are molecules from the Ingenuity database, inserted to connect all relevant molecules in a single network. Solid lines indicate known direct physical relationships between molecules, while dashed lines indicate known indirect functional relationships. Note the chemokine, CXCL11, and RSAD2 (viperin) are shown to be highly upregulated in this network, and were also associated with drusen in postmortem donor eyes [12]. The top two functionalities identified by Ingenuity for this molecular network are “Interferon Signaling,” “Role of Pattern Recognition Receptors in Recognition of Bacteria and Viruses”.

In our previous studies, we found that constituents of drusen such as amyloid beta and advanced glycation endproducts (AGE) are capable of activating the IFN-γ pathway [10,11]. AGE not only upregulated IFN-γ but also several of its downstream effectors including RSAD2, STAT1, CXCL10, and CXCL11 (Figures 1, 2 and3). Indeed, in postmortem human eyes, we found increased accumulation of RSAD2, CXCL10, and CXCL11 to be associated with the presence of drusen deposit [12]. Others have shown that *in vitro* stimulation of cultured RPE cells with IFN-γ led to polarized complement factor H (CFH) secretion predominantly localized to the apical surface [13–15]. This localization has been proposed to form a CFH gradient that could maintain retinal homeostasis and suppress a proinflammatory environment surrounding the photoreceptors. CFH is also a chemoattractant for monocytes [16]. In addition, when co-cultured with activated T cells, RPE cells produce an apical gradient of increased CCL7, CXCL9, CXCL10, and CXCL11 through T cell derived IFN-γ [5].

IFN-γ may be involved in AMD pathogenesis through macrophage polarization. Depending on the different microenvironment, macrophages can polarize into specific phenotypes, such as M1 or M2 macrophages [17]. The M2 subtype is predominantly pro-angiogenic, facilitating tissue repair and tends to increase during the normal aging process [18]. In contrast, the M1 subtype is predominantly proinflammatory and there is a pathological shift towards M1 subtype with the development of AMD. INF-γ can selectively promote polarization into M1 subtype [19]. M1 macrophages promote pathological inflammation through the secretion of proinflammatory cytokines such as IL-1β, IL-6, and TNF-α [20,21]. IL-1β is a strong proinflammatory factor. Along with IFN-γ, it synergistically increases the expression and secretion of IL-6, a potent inflammatory factor involved in the autoimmune and inflammatory disorders, in RPE cells [2]. Stimulation of human cells with IFN-γ potentiates IL-1β release and production [22].

Antibodies designed to block IFN-γ activity have been effective in the treatment of chronic inflammatory disorders such as rheumatoid arthritis and Crohn’s disease [23,24]. Antagonizing the IFN-γ pathway has been investigated in the context of AMD. Interferons are separated into three subtypes (type 1, 2, and 3) and differentiation between subtypes is based on the receptor through which they signal [25]. Type 1 interferon comprises of large sub-categories in humans including IFN-α, β, ε, κ and ω [26]. INF-γ is the only member of the type 2 subclass [25]. More recently, IFN-λ has been discovered and it is currently the sole member of the type 3 subclass [27]. Type 1 interferon including IFN-α and β have anti-proliferative and anti-angiogenic effects and has an antagonistic role to IFN-γ [28–32]. In the 1990s, IFN-α and β were used in the treatment of AMD [33–35]. It was found that IFN-α has minimal long-term therapeutic benefit and this was postulated to be due to the generation of anti-IFN-α antibodies as a result of treatment [36,37]. The utility of IFN-β was found to be more promising as it promotes proliferation and repair of damaged RPE and regression of CNV in monkey AMD models. More studies on the effectiveness of IFN-β have been published in literature [38–41]. Taken together, these studies indicate the importance of IFNs in AMD pathogenesis.

However, the use of IFN-γ as a therapeutic target can be complicated since in lower concentrations, IFN-γ shifts from being a proinflammatory factor to a more anti-inflammatory one [42,43]. At low levels, IFN-γ impedes homing of naïve T cells and Th2 cells to target organ [44]. Th2 cells induce fibrosis thereby counterbalancing the destructive effects of Th1 cells, which promote apoptosis [45,46]. Thus in the pathology of AMD, blocking IFN-γ may reduce the protective effects of Th2 and consequently aggravating the destructive function of Th1 cells [20,47].

## Is there any Beneficial Role of IFN-γ in Terms of Protective/Anti-inflammatory Effect?

The role of IFN-γ is complex, since IFN-γ is associated with both protective and destructive inflammatory processes. IFN-γ is classically considered as a pro-inflammatory factor, yet in recent years, multiple studies have found IFN-γ to mediate an immune-modulatory and protective function. For example, in human endothelial cells IFN-γ inhibits the angiogenic activity of VEGF through activation of STAT1 pathway [48], down-regulating VEGF mRNA in a dosage-dependent manner [49]. This may help to inhibit excess angiogenesis process in wet AMD. Interestingly, another study suggests that IFN-γ is able to mediate VEGF upregulation in RPE cells through the PI-3K/Akt/mTOR/p70 S6 kinase pathway, and is independent of STAT1 [50]. Therefore, IFN-γ associated STAT1 activation may be beneficial. Another piece of evidence comes from the study of IFN-γ up-regulating CFH expression in RPE cells [15]. CFH can keep the complement cascade in check and prevent tissue injury from excessive complement activation [51]. CFH is transcriptionally upregulated by STAT1, but oxidative stress, one of the most important risk factors for AMD, can disrupt this process by acetylating FOXO3, which competes with STAT1 for binding to the CFH promoter [15,52]. It is also known that STAT1-deficient mouse are highly susceptible to autoimmune disorders [53] and given that AMD may be considered an autoimmune disease [54,55], preserving STAT1 activation by IFN-γ may be important in mitigating AMD progression. Furthermore, IFN-γ can tilt the balance toward STAT1 by deactivating STAT3. STAT1 and STAT3 are negative regulators of each other and activate distinctly different downstream pathways [56]. STAT1 plays a key role in inhibiting angiogenesis, while STAT3 induces the production of VEGF directly or indirectly through hypoxia-inducible factor 1α in tumor cells [57-61]. INF-γ deactivates STAT3 by promoting STAT3 dephosphorylation [62]. Topical IFN-γ is being investigated for as a means of treatment for macular edema in uveitis (http://clinicaltrials.gov/show/NCT00943982).

IFN-γ further down-regulates the VEGF pathway through the up-regulation IL-1RA [42]. IL-1RA inhibits IL-1 receptor bindings to IL-1, and thus performs an anti-inflammatory function [42]. IL-1β is strongly implicated in the pathogenesis of chronic inflammatory diseases [63]. Indeed, human RPE cells treated with amyloid beta strongly upregulated IL-1β [10]. Aberrant auto-upregulation of IL- 1β leads to excessive inflammation and promotes angiogenesis through upregulation of VEGF [64]. IL-1β is capable of inducing reactive oxygen species (ROS) in RPE cells [65] and ROS triggers the release of IL-8, which recruits pro-inflammatory cells such as macrophages [10,65,66]. With macrophages present in drusen deposits of AMD eyes, it plays a key role in promoting neovascular proliferation [67,68]. In AMD models IL-1RA have been shown to be effective in reducing the degree of CNV formation, likely through the inhibition of IL-1 pathways [69].

IFN-γ may also play a beneficial role by regulating Th17 cells. Th17 cells have been characterized as a subclass of T cells and implicated in numerous autoimmune disorders including diabetes, autoimmune encephalomyelitis, autoimmune uveitis, and thyroiditis [43]. Recently Th17 cell specific cytokines, IL-17 and IL-22 are found to be elevated in serum of AMD patients, further implicating Th17 cells in the pathogenesis of AMD [70]. The upregulation of IL-17 is believed to be mediated by complement activation product C5a [70]. In AMD patients, C5a is elevated in serum and may be associated with AMD at risk gene variant, which regulates complement activation [51,71]. IFN-γ inhibits T cell differentiation into Th17 and in murine models of Th1 related autoimmune disorders, knocking out IFN-γ results in a more severe disease process. This worsening is believed to be mediated through Th17 cell [72–74].

## Conclusion

In conclusion, IFN-γ plays an intriguing role in the pathogenesis of AMD. Certainly, several lines of evidence suggest that inhibition of IFN-γ may prevent inflammation-mediated responses that contribute to the progression of AMD. However, given the evidence suggesting its involvement in anti-inflammatory and neuroprotective mechanisms in a number of murine autoimmune disease models [31], it is still debatable whether therapeutic inhibition of IFN-γ pathways would help counteract the progression of AMD. Further characterization of the IFN-γ mediated immunomodulatory pathways that are involved in the pathogenesis of AMD is necessary.

## Figures and Tables

**Figure 3:**
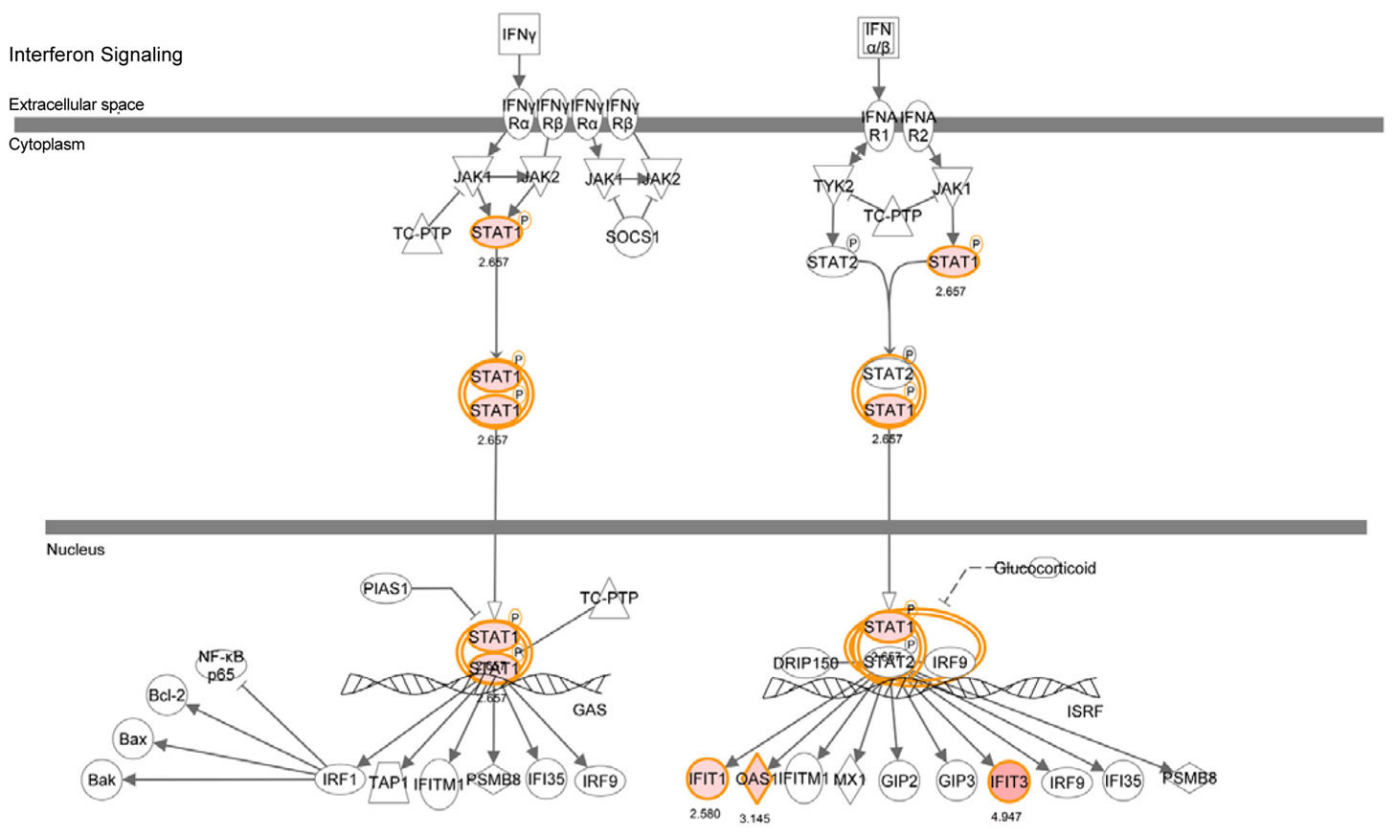
The Interferon Signaling Pathway identified by Ingenuity software. This is one of the canonical pathways that contain statistically significantly more genes than expected by chance in the group of 41-up and 18-down regulated RPE genes in response to AGE stimulation in vitro. Red symbols represent the genes from our stimulation study, while the white symbols represent genes inserted by Ingenuity to connect relevant molecules into a single pathway.

## References

[1] Resnikoff S, Pascolini D, Etya’ale D, Kocur I, Pararajasegaram R (2004). Global data on visual impairment in the year 2002. Bull World Health Organ.

[2] Nagineni CN, Detrick B, Hooks JJ (1994). Synergistic effects of gamma interferon on inflammatory mediators that induce interleukin-6 gene expression and secretion by human retinal pigment epithelial cells. Clin Diagn Lab Immunol.

[3] Chakrabarty P, Ceballos-Diaz C, Beccard A, Janus C, Dickson D (2010). IFN-gamma promotes complement expression and attenuates amyloid plaque deposition in amyloid beta precursor protein transgenic mice. J Immunol.

[4] Huang Y, Krein PM, Winston BW (2001). Characterization of IFN-gamma regulation of the complement factor B gene in macrophages. Eur J Immunol.

[5] Juel HB, Faber C, Udsen MS, Folkersen L, Nissen MH (2012). Chemokine expression in retinal pigment epithelial ARPE-19 cells in response to coculture with activated T cells. Invest Ophthalmol Vis Sci.

[6] Dastgheib K, Green WR (1994). Granulomatous reaction to Bruch’s membrane in age-related macular degeneration. Arch Ophthalmol.

[7] Penfold PL, Wong JG, Gyory J, Billson FA (2001). Effects of triamcinolone acetonide on microglial morphology and quantitative expression of MHC-II in exudative age-related macular degeneration. Clin Experiment Ophthalmol.

[8] Ding X, Patel M, Chan CC (2009). Molecular pathology of age-related macular degeneration. Prog Retin Eye Res.

[9] Roque RS, Rosales AA, Jingjing L, Agarwal N, Al-Ubaidi MR (1999). Retina-derived microglial cells induce photoreceptor cell death in vitro. Brain Res.

[10] Kurji KH, Cui JZ, Lin T, Harriman D, Prasad SS (2010). Microarray analysis identifies changes in inflammatory gene expression in response to amyloid-beta stimulation of cultured human retinal pigment epithelial cells. Invest Ophthalmol Vis Sci.

[11] Lin T, Cui J, Matsubara J (2012). Advance glycation end product and its role in age-related degenerative diseases of the eye.

[12] Jiang K, To E, Cui JZ, Cao S, Gao J (2012). Drusen and pro-inflammatory mediators in the post-mortem human eye. J Clinic Experiment Ophthalmol.

[13] Kim YH, He S, Kase S, Kitamura M, Ryan SJ (2009). Regulated secretion of complement factor H by RPE and its role in RPE migration. Graefes Arch Clin Exp Ophthalmol.

[14] Lau LI, Chiou SH, Liu CJ, Yen MY, Wei YH (2011). The effect of photo-oxidative stress and inflammatory cytokine on complement factor H expression in retinal pigment epithelial cells. Invest Ophthalmol Vis Sci.

[15] Wu Z, Lauer TW, Sick A, Hackett SF, Campochiaro PA (2007). Oxidative stress modulates complement factor H expression in retinal pigmented epithelial cells by acetylation of FOXO3. J Biol Chem.

[16] Nabil K, Rihn B, Jaurand MC, Vignaud JM, Ripoche J (1997). Identification of human complement factor H as a chemotactic protein for monocytes. Biochem J.

[17] Lawrence T, Natoli G (2011). Transcriptional regulation of macrophage polarization: enabling diversity with identity. Nat Rev Immunol.

[18] Cousins SW, Espinosa-Heidmann DG, Miller DM, Pereira-Simon S, Hernandez EP (2012). Macrophage activation associated with chronic murine cytomegalovirus infection results in more severe experimental choroidal neovascularization. PLoS Pathog.

[19] Brown J, Wallet MA, Krastins B, Sarracino D, Goodenow MM (2010). Proteome bioprofiles distinguish between M1 priming and activation states in human macrophages. J Leukoc Biol.

[20] Cao X, Shen D, Patel MM, Tuo J, Johnson TM (2011). Macrophage polarization in the maculae of age-related macular degeneration: a pilot study. Pathol Int.

[21] Cousins SW, Espinosa-Heidmann DG, Csaky KG (2004). Monocyte activation in patients with age-related macular degeneration: a biomarker of risk for choroidal neovascularization?. Arch Ophthalmol.

[22] Masters SL, Mielke LA, Cornish AL, Sutton CE, O’Donnell J (2010). Regulation of interleukin-1beta by interferon-gamma is species specific, limited by suppressor of cytokine signalling 1 and influences interleukin-17 production. EMBO Rep.

[23] Vacchelli E, Galluzzi L, Eggermont A, Galon J, Tartour E (2012). Trial Watch: Immunostimulatory cytokines. Oncoimmunology.

[24] Hommes DW, Mikhajlova TL, Stoinov S, Stimac D, Vucelic B (2006). Fontolizumab, a humanised anti-interferon gamma antibody, demonstrates safety and clinical activity in patients with moderate to severe Crohn’s disease. Gut.

[25] Platanias LC (2005). Mechanisms of type-I- and type-II-interferon-mediated signalling. Nat Rev Immunol.

[26] Pestka S, Krause CD, Walter MR (2004). Interferons, interferon-like cytokines, and their receptors. Immunol Rev.

[27] Kotenko SV, Gallagher G, Baurin VV, Lewis-Antes A, Shen M (2003). IFN-lambdas mediate antiviral protection through a distinct class II cytokine receptor complex. Nat Immunol.

[28] Roberts ZJ, Ching LM, Vogel SN (2008). IFN-beta-dependent inhibition of tumor growth by the vascular disrupting agent 5,6-dimethylxanthenone-4-acetic acid (DMXAA). J Interferon Cytokine Res.

[29] Fultz MJ, Vogel SN (1998). Analysis of the antagonist effect of IFN-alpha on IFN-gamma-induced interferon consensus sequence binding protein messenger RNA in murine macrophages. J Inflamm.

[30] Li R, Maminishkis A, Wang FE, Miller SS (2007). PDGF-C and -D induced proliferation/migration of human RPE is abolished by inflammatory cytokines. Invest Ophthalmol Vis Sci.

[31] Borden EC, Hogan TF, Voelkel JG (1982). Comparative antiproliferative activity in vitro of natural interferons alpha and beta for diploid and transformed human cells. Cancer Res.

[32] Indraccolo S (2010). Interferon-alpha as angiogenesis inhibitor: learning from tumor models. Autoimmunity.

[33] Tobe T, Takahashi K, Kishimoto N, Ohkuma H, Uyama M (1995). Effects of interferon-beta on repair of the retinal pigment epithelium after laser photocoagulation. Nihon Ganka Gakkai Zasshi.

[34] Tobe T, Takahashi K, Ohkuma H, Uyama M (1995). The effect of interferon-beta on experimental choroidal neovascularization. Nihon Ganka Gakkai Zasshi.

[35] Engler CB, Sander B, Koefoed P, Larsen M, Vinding T (1993). Interferon alpha-2a treatment of patients with subfoveal neovascular macular degeneration. A pilot investigation. Acta Ophthalmol (Copenh).

[36] Poliner LS, Tornambe PE, Michelson PE, Heitzmann JG (1993). Interferon alpha-2a for subfoveal neovascularization in age-related macular degeneration. Ophthalmology.

[37] Ross C, Engler CB, Sander B, Bendtzen K (2002). IFN-alpha antibodies in patients with age-related macular degeneration treated with recombinant human IFN-alpha2a. J Interferon Cytokine Res.

[38] Qiao H, Sakamoto T, Hinton DR, Gopalakrishna R, Ishibashi T (2001). Interferon beta affects retinal pigment epithelial cell proliferation via protein kinase C pathways. Ophthalmologica.

[39] Kimoto T, Takahashi K, Tobe T, Fujimoto K, Uyama M (2002). Effects of local administration of interferon-beta on proliferation of retinal pigment epithelium in rabbit after laser photocoagulation. Jpn J Ophthalmol.

[40] Yasukawa T, Kimura H, Tabata Y, Kamizuru H, Miyamoto H (2002). Targeting of interferon to choroidal neovascularization by use of dextran and metal coordination. Invest Ophthalmol Vis Sci.

[41] Hooks JJ, Nagineni CN, Hooper LC, Hayashi K, Detrick B (2008). IFN-beta provides immuno-protection in the retina by inhibiting ICAM-1 and CXCL9 in retinal pigment epithelial cells. J Immunol.

[42] Mühl H, Pfeilschifter J (2003). Anti-inflammatory properties of pro-inflammatory interferon-gamma. Int Immunopharmacol.

[43] Kelchtermans H, Billiau A, Matthys P (2008). How interferon-gamma keeps autoimmune diseases in check. Trends Immunol.

[44] Flaishon L, Topilski I, Shoseyov D, Hershkoviz R, Fireman E (2002). Cutting edge: anti-inflammatory properties of low levels of IFN-gamma. J Immunol.

[45] Caspi RR (2002). Th1 and Th2 responses in pathogenesis and regulation of experimental autoimmune uveoretinitis. Int Rev Immunol.

[46] Wynn TA (2004). Fibrotic disease and the T(H)1/T(H)2 paradigm. Nat Rev Immunol.

[47] Giunta B, Fernandez F, Nikolic WV, Obregon D, Rrapo E (2008). Inflammaging as a prodrome to Alzheimer’s disease. J Neuroinflammation.

[48] Battle TE, Lynch RA, Frank DA (2006). Signal transducer and activator of transcription 1 activation in endothelial cells is a negative regulator of angiogenesis. Cancer Res.

[49] Kawano Y, Matsui N, Kamihigashi S, Narahara H, Miyakawa I (2000). Effects of interferon-gamma on secretion of vascular endothelial growth factor by endometrial stromal cells. Am J Reprod Immunol.

[50] Liu B, Faia L, Hu M, Nussenblatt RB (2010). Pro-angiogenic effect of IFNgamma is dependent on the PI3K/mTOR/translational pathway in human retinal pigmented epithelial cells. Mol Vis.

[51] Patel M, Chan CC (2008). Immunopathological aspects of age-related macular degeneration. Semin Immunopathol.

[52] Beatty S, Koh H, Phil M, Henson D, Boulton M (2000). The role of oxidative stress in the pathogenesis of age-related macular degeneration. Surv Ophthalmol.

[53] Nishibori T, Tanabe Y, Su L, David M (2004). Impaired development of CD4+ CD25+ regulatory T cells in the absence of STAT1: increased susceptibility to autoimmune disease. J Exp Med.

[54] Iannaccone A, Neeli I, Krishnamurthy P, Lenchik NI, Wan H (2012). Autoimmune biomarkers in age-related macular degeneration: a possible role player in disease development and progression. Adv Exp Med Biol.

[55] Morohoshi K, Goodwin AM, Ohbayashi M, Ono SJ (2009). Autoimmunity in retinal degeneration: autoimmune retinopathy and age-related macular degeneration. J Autoimmun.

[56] Ho HH, Ivashkiv LB (2006). Role of STAT3 in type I interferon responses. Negative regulation of STAT1-dependent inflammatory gene activation. J Biol Chem.

[57] Stephanou A, Latchman DS (2003). STAT-1: a novel regulator of apoptosis. Int J Exp Pathol.

[58] Levy DE, Lee CK (2002). What does Stat3 do?. J Clin Invest.

[59] Kortylewski M, Jove R, Yu H (2005). Targeting STAT3 affects melanoma on multiple fronts. Cancer Metastasis Rev.

[60] Shin J, Lee HJ, Jung DB, Jung JH, Lee HJ (2011). Suppression of STAT3 and HIF-1 alpha mediates anti-angiogenic activity of betulinic acid in hypoxic PC-3 prostate cancer cells. PLoS One.

[61] Xu Q, Briggs J, Park S, Niu G, Kortylewski M (2005). Targeting Stat3 blocks both HIF-1 and VEGF expression induced by multiple oncogenic growth signaling pathways. Oncogene.

[62] Fang P, Hwa V, Rosenfeld RG (2006). Interferon-gamma-induced dephosphorylation of STAT3 and apoptosis are dependent on the mTOR pathway. Exp Cell Res.

[63] Dinarello CA (2010). IL-1: discoveries, controversies and future directions. Eur J Immunol.

[64] Jung YD, Liu W, Reinmuth N, Ahmad SA, Fan F (2001). Vascular endothelial growth factor is upregulated by interleukin-1 beta in human vascular smooth muscle cells via the P38 mitogen-activated protein kinase pathway. Angiogenesis.

[65] Yang D, Elner SG, Bian ZM, Till GO, Petty HR (2007). Pro-inflammatory cytokines increase reactive oxygen species through mitochondria and NADPH oxidase in cultured RPE cells. Exp Eye Res.

[66] Hwang YS, Jeong M, Park JS, Kim MH, Lee DB (2004). Interleukin-1beta stimulates IL-8 expression through MAP kinase and ROS signaling in human gastric carcinoma cells. Oncogene.

[67] Espinosa-Heidmann DG, Suner IJ, Hernandez EP, Monroy D, Csaky KG (2003). Macrophage depletion diminishes lesion size and severity in experimental choroidal neovascularization. Invest Ophthalmol Vis Sci.

[68] Jager MJ, Klaver CC (2007). Macrophages feel their age in macular degeneration. J Clin Invest.

[69] Olson JL, Courtney RJ, Rouhani B, Mandava N, Dinarello CA (2009). Intravitreal anakinra inhibits choroidal neovascular membrane growth in a rat model. Ocul Immunol Inflamm.

[70] Liu B, Wei L, Meyerle C, Tuo J, Sen HN (2011). Complement component C5a promotes expression of IL-22 and IL-17 from human T cells and its implication in age-related macular degeneration. J Transl Med.

[71] Klein RJ, Zeiss C, Chew EY, Tsai JY, Sackler RS (2005). Complement factor H polymorphism in age-related macular degeneration. Science.

[72] Harrington LE, Hatton RD, Mangan PR, Turner H, Murphy TL (2005). Interleukin 17-producing CD4+ effector T cells develop via a lineage distinct from the T helper type 1 and 2 lineages. Nat Immunol.

[73] Newman AM, Gallo NB, Hancox LS, Miller NJ, Radeke CM (2012). Systems-level analysis of age-related macular degeneration reveals global biomarkers and phenotype-specific functional networks. Genome Med.

[74] Damsker JM, Hansen AM, Caspi RR (2010). Th1 and Th17 cells: adversaries and collaborators. Ann N Y Acad Sci.

